# Epilepsy and psychosis– where are we now?

**DOI:** 10.1192/j.eurpsy.2021.1378

**Published:** 2021-08-13

**Authors:** I. Pereira, V. Nogueira, J. Teixeira

**Affiliations:** Clínica 4 - Unidade De Alcoologia E Novas Dependências, Centro Hospitalar Psiquiátrico de Lisboa, Lisboa, Portugal

**Keywords:** ictal psychosis, epileptic psyhosis

## Abstract

**Introduction:**

Epilepsy has long been considered a risk factor for psychosis, and studies estimate that up to 80% of patients with epilepsy will experience a psychotic episode at some point in the course of their disease. However, data on the treatment of psychotic disorders in epilepsy is limited and the management of these problems is still founded on individual clinical experience.

**Objectives:**

To assess evidence pertaining psychosis related to epilepsy, especially its risks factors and treatment approaches available.

**Methods:**

Bibliographic research was made through the PubMed/NCBI database. No time limit was specified on the search. Pertinent manuscripts were individually reviewed for additional relevant citations.

**Results:**

Recent literature shows a prevalence in psychotic disorders of 5.6%, and up to 7% in patients with epilepsy. So far, mechanisms of psychosis in epileptic patients remain unknown. Risk factors are earlier age of epilepsy onset, more frequent seizures, longer duration of epilepsy, high number of relatives with epilepsy and long-term antiepileptic drugs therapy. Psychiatric manifestations may include both positive and negative symptoms, including auditory hallucinations, paranoid delusions, and disorganized thought and/or behaviour. Poor adherence to treatment with oral antipsychotics occurs in more than 40% of patients; long-acting injectable medication should be considered, bearing in mind interactions with anti-epileptic medication and possibility of increased side effects.

**Conclusions:**

Our findings emphasize the importance of early recognition and management of psychosis in epileptic patients. Unfortunately, there is lack of evidence for the use of antipsychotic medication in epileptic patients, since available studies pertain to populations with primary psychiatric disorders.

